# Immunization route-mediated differences in long-term maturation of humoral immune response induced by adenovirus vector-based COVID-19 vaccine Sputnik V in nonhuman primates

**DOI:** 10.3389/fimmu.2025.1634187

**Published:** 2025-09-12

**Authors:** Amir I. Tukhvatulin, Ilya V. Gordeychuk, Alina S. Dzharullaeva, Inna V. Dolzhikova, Ekaterina O. Bayurova, Fatima M. Izhaeva, Anna V. Kovyrshina, Alla S. Zhitkevich, Daria V. Avdoshina, Stanislav A. Gulyaev, Tatiana V. Gulyaeva, Andrey V. Moroz, Ilias B. Esmagambetov, Ilya D. Zorkov, Anna A. Iliukhina, Artem Y. Shelkov, Alina S. Erokhova, Dmitry V. Shcheblyakov, Olga V. Zubkova, Aydar A. Ishmukhametov, Denis Y. Logunov, Alexander L. Gintsburg

**Affiliations:** ^1^ National Research Center for Epidemiology and Microbiology Named after Honorary Academician N.F. Gamaleya of the Ministry of Health of the Russian Federation, Moscow, Russia; ^2^ Chumakov Federal Scientific Center for Research and Development of Immune-and-Biological Products of Russian Academy of Sciences (Institute of Poliomyelitis), Moscow, Russia; ^3^ I.M. Sechenov First Moscow State Medical University of the Ministry of Health of the Russian Federation (Sechenov University), Moscow, Russia

**Keywords:** COVID-19, SARS-CoV-2, Sputnik V vaccine, common marmosets, serum maturation, long-term immunogenicity

## Abstract

**Introduction:**

On the background of kaleidoscopic changes of SARS-CoV-2 circulating variants, constant presence of SARS-CoV-2 in the human population hampers the dissection of native long-term immunogenicity of COVID-19 vaccines.

**Methods:**

For this purpose, we performed a more than two-year-long evaluation of parameters of the humoral immune response elicited by intramuscularly (IM) and intranasally (IN) delivered adenovirus vector-based Sputnik V vaccine in nonhuman primates (NHP, Common marmosets), which are naturally nonsusceptible for SARS-CoV-2 infection.

**Results:**

Although both immunization routes elicited prominent humoral immune responses in a short-term perspective, the long-term kinetics significantly differed between the IM and IN groups. While the titers of local and systemic antigen-specific antibodies (both IgA and IgG) nearly disappeared within two years upon IN vaccination, IM vaccination led to the highest IgG values in nasal swabs as well as IgA and IgG in serum specimens from NHPs by the end of observation period (day 764). Unlike IN vaccination, IM vaccination also resulted in a continuous long-term increase in serum maturation parameters such as antibody avidity, neutralization potency and breadth.

**Discussion:**

The present study provides valuable information about distinct features of the long-term postvaccination humoral immune response in nonhuman primates induced by adenoviral COVID-19 vaccine administered by the intramuscular and intranasal routes commonly used in clinical practice.

## 1 Introduction

Since the onset of the pandemic caused by SARS-CoV-2 and the subsequent imminent rollout of COVID-19 vaccines, the question regarding time-dependent changes in post-vaccination humoral immune response, including the potential for cross-neutralization of newly emerging variants of concern (VOCs), has been pivotal for the vaccine development.

While more than 40 original COVID-19 vaccines have been authorized for clinical use, only a few longitudinal studies described the long-term changes in immunity after COVID-19 vaccination (1). Furthermore, the published data refer to vaccines administered intramuscularly only, although at least 16 intranasally administered vaccines have been tested in clinical trials, including 4 licensed ones (Razi-Cov-Pars, Convidecia, iNCOVACC, and ChAdOx1) (2).

Longitudinal studies showed that both mRNA-based COVID-19 vaccines BNT162b2 (Pfizer/BioNTech) and mRNA-1273 (Moderna) result in serum avidity maturation and extension of cross-neutralization breadth, while titers of anti-Spike IgG and neutralizing antibodies wane within several months after immunization (3–5). The mechanism underlying the observed findings, termed as antibody affinity maturation, is based on selection and expansion of B cells with higher-affinity antibodies (along with broadening of their neutralizing activity) in germinal centers (GCs) upon prolonged antigen presentation (6).

Much less is known about changes in maturation parameters of humoral immunity using COVID-19 vaccines based on other platforms (live-attenuated, inactivated, protein, viral-vectored ones, etc.). Studies addressing the long-term kinetics of antigen-binding and virus-neutralizing antibodies elicited by the COVID-19 vaccine based on replication-incompetent recombinant adenovirus type 26 (Ad26), Ad26.COV2.S (Janssen Vaccines), have yielded contradictory results. Having compared antibody responses between the peak immunity at four weeks and eight months after administration of one-dose Ad26.COV2.S vaccine, Ai-ris Y. Collier at al. showed that the titers of live-virus neutralizing antibodies increased (from 146 to 629), while the receptor-binding domain (RBD)-specific IgG titers declined over this time (from 1361 to 843) (7). On the contrary, another study detected an elevation of RBD-binding IgG titers (from 645 on day 29 to 1306 on day 239) together with a downward trend in the pseudovirus neutralizing antibody titer (from 272 to 192) after the same eight months after single administration of Ad26.COV2.S vaccine (8). The third longitude study reported a decrease in pseudotyped virus neutralization ability (from 105 to 41) with a non-significant mild decline in RBD-binding IgG titers (from 20,447 to 15,379) in vaccinees’ serum observed between 1.5 and 6 months upon single-dose Ad.26.COV.2 vaccination (9). As none of the currently available COVID-19 vaccines provide absolute protection against SARS-CoV-2 infection, prolonged observation of clinical trial participants is associated with an increased risk of SARS-CoV-2 infection (10). In this regard, such inconsistence might be associated with unregistered SARS-CoV-2 infections during observation period, which is known to cause significant changes in immune response (11, 12). Therefore, the dissection of COVID-19 uninfected vaccinees (especially using small sample size cohorts) is getting of even greater concern in longitudinal studies compared to short-term ones.

Summarizing the above, several questions currently remain to be clarified: (i) whether other COVID-19 vaccines based on platforms alternative to mRNA could result in maturation of humoral immune response and (ii) how the vaccination route affects serum maturation parameters.

Considering these points, the present article aimed to investigate the long-term humoral immune responses (up to postvaccination day 764) using adenovirus vector-based prime-boost Gam-COVID-Vac (Sputnik V) vaccine after intramuscular and intranasal administration in nonhuman primates (common marmosets), which are naturally nonsusceptible to SARS-CoV-2 infection (13). Additionally, in order to verify the absence of occasional viral exposure, the animals were tested for anti-N IgG antibodies during the entire investigation period. This strategy allows one to observe the time-dependent evolution of humoral responses in the context of exclusion of unregistered immune boosts.

## 2 Materials and methods

### 2.1 Animals

C57BL/6 female SPF mice (4–5 weeks old) were procured from an onsite animal breeding facility at the N.F. Gamaleya National Research Center for Epidemiology and Microbiology. Mice were housed in autoclaved ventilated ISOCage P systems (Techniplast, Italy) containing sterile drinking water, corn bedding, and standard chow diet.

Healthy adult 2–6-year-old common marmosets (at the beginning of study) were bred and maintained in the Experimental Clinic of Callitrichidae at the Chumakov Federal Scientific Center for Research and Development of Immune-and-Biological Products of Russian Academy of Sciences. The primates were kept according to the EU Directive 2010/63/EU and the Russian sanitary regulations for experimental animal clinics (1045-73).

### 2.2 Immunization and further sampling procedures

Marmosets were subsequently immunized with two-component vaccine Sputnik V (Gam-COVID-Vac) consisting of recombinant adenovirus type 26 (component 1) and recombinant adenovirus type 5 (component 2) with 24-day interval. The first group was intramuscularly injected with 1/5 of the human dose (2 × 10^10^vp, 100µl) of Sputnik V vaccine into the femoral muscles. The second group was inoculated intranasally with 50 µL of the Sputnik V vaccine into each nostril with an automatic pipette (a total of 2 × 10^10^vp). On the same days, animals from the placebo group were injected with the same volume of sterile isotonic saline preparation via both the IN and IM routes. Marmoset blood samples were collected at specified time points from restrained conscious animals by femoral vein puncture. Nasal swabs were collected using type A human urinogenital swabs (Polimernye Izdeliya Company, Russia) placed in 0.1 mL PBS containing proteinase inhibitor cocktail (Sigma-Aldrich) and 0.1% m/w sodium azide. The procedures did not cause any significant distress to the animals due to the preceding training.

All the animals before vaccination (day 0) and during the observation period tested negative for anti-N IgG antibodies using Vector-Best Elisa plates (Russia) as a source of the N protein and anti-monkey IgG (Sigma Aldrich, USA) as detection antibodies.

### 2.3 Cell lines and viruses

Vero E6 (ATCC CRL-1586) cells were maintained in Dulbecco’s modified Eagle medium (DMEM, HyClone, Cytiva, USA) supplemented with 10% or 2% heat-inactivated fetal bovine serum (FBS, Capricorn Scientific, Germany), L-glutamine (4 mM), and penicillin/streptomycin solution (100 IU/mL; 100 μg/mL) (PanEco, Moscow, Russia). The SARS-CoV-2 strain B.1.1.1 or PMVL-1 (GISAID EPI_ISL_421275), B.1.617.2 (Delta) and B.1.1.529.5 (Omicron) initially isolated from a nasopharyngeal swab was obtained from the State Collection of Viruses of the Gamaleya National Research Center for Epidemiology and Microbiology in Moscow and used in titration of NtAbs studies. Isolation and further propagation were performed in Vero E6 cells in DMEM (HyClone Cytiva, Austria) supplemented with 2% heat-inactivated FBS (Capricorn Scientific GmbH, Germany): the cells were infected at multiplicity of infection (MOI)  =  0.01 and incubated at 37°C in 5% CO_2_. The culture medium was collected at 72 h and clarified by centrifugation at 9000 g for 10 min at +4°C. The culture medium containing the virus was aliquoted, frozen, and stored at −80°C.

### 2.4 Evaluation of antibody titers in biological samples

RBD (from Wuhan-Hu-1) pre-coated 96-well plates from the ELISA kit developed at the Gamaleya National Research Center for Epidemiology and Microbiology and approved for clinical use in Russia (RZN 2020/10393 2020-05-18) were used for detecting SARS-CoV-2 RBD-specific antibodies. In the case of anti-S IgG antibodies, recombinant S proteins from Wuhan-Hu-1, B1.617.2 and B1.1.529.5 (Sino Biological, China) were used for coating 96-well plates (SPL, South Korea). Murine antigen-specific antibodies in the obtained samples were detected using anti-mouse total IgG secondary HRP-conjugated antibodies (used in 1:5000 dilution) purchased from Abcam (UK). Anti-monkey IgG (Sigma Aldrich, USA, 1:20,000 dilution) and anti-human IgA (clone 3B4, a gift from Dr. Marina Samoylovich, 1:40,000 dilution) secondary antibodies, both conjugated to HRP, were used to evaluate the humoral response of marmosets to vaccination. The clone 3B4 recognizes alpha-1 and alpha-2 heavy chains of human IgA presented in both monomeric and polymeric forms. The ELISA protocol was used according to the procedure published earlier (14). The colorimetric signal was measured at 450 nm using a Multiscan FC spectrophotometric plate reader (Thermo Fisher Scientific, Waltham, MA, USA) 30 min after adding the stop solution (4 M H_2_SO_4_).

### 2.5 Avidity enzyme-linked immunosorbent assay

The same RBD or S pre-coated ELISA 96-well plates were used for antibody avidity evaluation. After 1-hr incubation with serum, equal volumes of denaturing solution (PBS supplemented with 8M urea) and control phosphate-buffered saline were added to the wells for 10 min. The next steps (washing with PBS/Tween 0.05%, incubation with secondary anti-monkey or anti-mouse IgG antibodies, addition of TMB and subsequently, H_2_SO_4_ stopping solution) were done identically as described above for anti-RBD IgG ELISA. The avidity index (AI, %) for each serum sample was calculated as the ratio between the OD of the well containing a denaturing solution and the OD of the well containing the control buffer at the same serum dilution (15).

### 2.6 Neutralization assay with live SARS-CoV-2

Samples were inactivated by incubation at 56°C for 30 min prior to twofold serial dilution in complete DMEM supplemented with 2% heat-inactivated FBS. The samples were then mixed at a 1:1 ratio with 100TCID50 (50% tissue culture infectious dose) of SARS-CoV-2 variants in total volume of 100 μL and incubated at 37°C for 1 hr. The antibody–virus complexes were then added to Vero E6 cell monolayer and incubated for 96 hrs. The cytopathic effect (CPE) of the virus on the cells was assessed visually. Neutralization titer was defined as the highest serum dilution without any CPE in two of three replicable wells. Samples with no neutralization at starting dilution points were attributed to twofold lower values.

### 2.7 Statistical analysis

The data obtained were analyzed using the GraphPad Prizm software 10.2 and Microsoft Office Excel 2023.

## 3 Results

### 3.1 Intramuscular and intranasal immunization with Sputnik V vaccine results in different long-term kinetics of local IgA and IgG responses in nonhuman primates

Our previous study reported the differences in short-term (up to day 116) local and systemic humoral responses in common marmosets after IN immunization compared to the original IM route of immunization with Sputnik V vaccine (14). As no peak levels of antigen-binding IgA and IgG antibodies were detected within the initial observation period, we continued collecting animal nasal swabs and blood samples on days 462 and 764, as well as evaluated antibody maturation parameters for different immunization routes.

As the RBD of the SARS-CoV-2 spike protein binds to the ACE2 receptor on host cells to facilitate viral entry, we measured anti-RBD antibodies, which had been shown to be one of the correlates of protection against SARS-CoV-2 (16).

**Figure 1:**
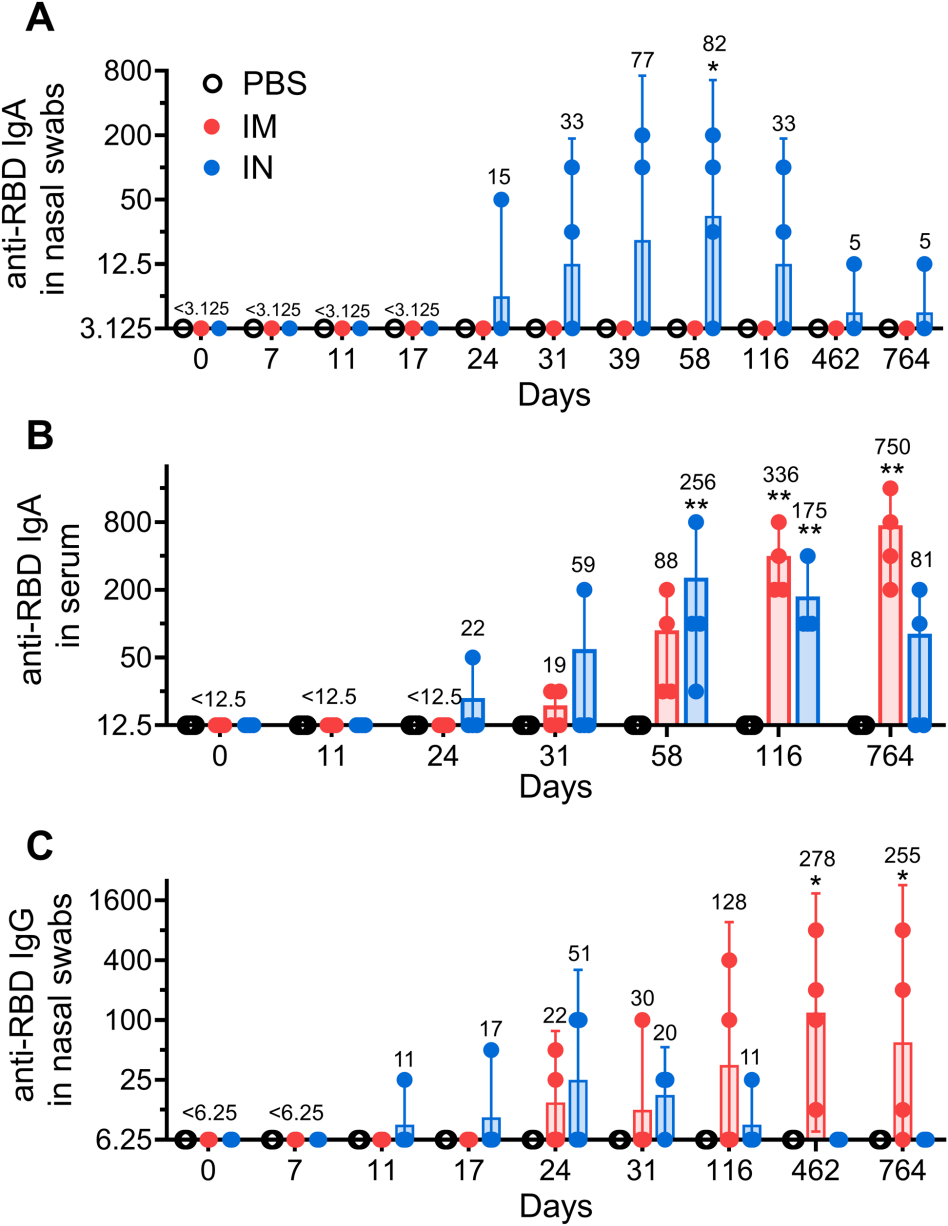
The long-term local humoral immune response in common marmosets after intramuscular (IM) or intranasal (IN) vaccination. Receptor-binding domain (RBD) specific IgA antibodies were measured in nasal swabs **(A)** as well as in blood serum **(B)**. Locally secreted anti-RBD IgGs **(C)** were measured in nasal swabs. NHPs were vaccinated twice with Sputnik V vaccine (2 × 10^10^ vp) intramuscularly (IM) or intranasally (IN) with a 24-day interval. The placebo group received PBS via both the IM and IN route on the same days. Bars represent the geometric mean for each group with 95% CI. The Geometric Mean Reciprocal Titers (GMRT) are presented above each group. Dots show individual data points. Significant differences between chosen day and day 0 (before vaccination) are shown above the bars with asterisks (*p < 0.05, **p < 0.01, non-parametric paired Friedman test).

An analysis of local humoral immune responses showed that only IN vaccination induced significant secretory anti-RBD IgA antibodies in nasal swabs. The Geometric Mean Reciprocal Titer (GMRT) of anti-RBD IgA peaked on day 58 after the first vaccination (GMRT 82) and subsequently vanished by day 462, supporting the notion that vaccine-induced local IgA response has limited durability (**Figure 1A**) (17). Similar to the local IgA, serum anti-RBD IgA antibodies upon IN vaccination briefly peaked on day 58 (GMRT 256) and began to decrease at late time points, reaching an insignificant level compared to the PBS-treated group on day 764 (**Figure 1B**). IN vaccination showed no induction of local IgG antibodies (**Figure 1C**).

While IM vaccination elicited no detectable levels of IgA antibodies in nasal swabs, titers of anti-RBD IgA antibodies in animal serum as well as titers of IgG antibodies in nasal swabs were constantly rising over time to reach GMRT 750 and 255, respectively, on day 764. To account for possible varability in the sampling procedure, we also measured total IgG levels on days 7, 116 and 764 in nasal swabs (18). We found even more prominent time-dependent increase in anti-RBD IgG levels after normalization to the total IgG concentration (both expressed in mcg/mL) upon IM vaccination (**Supplementary Figure S1**).

### 3.2 Intramuscular administration of Sputnik V vaccine results in a more potent, durable and mature systemic humoral immune response in nonhuman primates compared to intranasal administration

**Figure 2:**
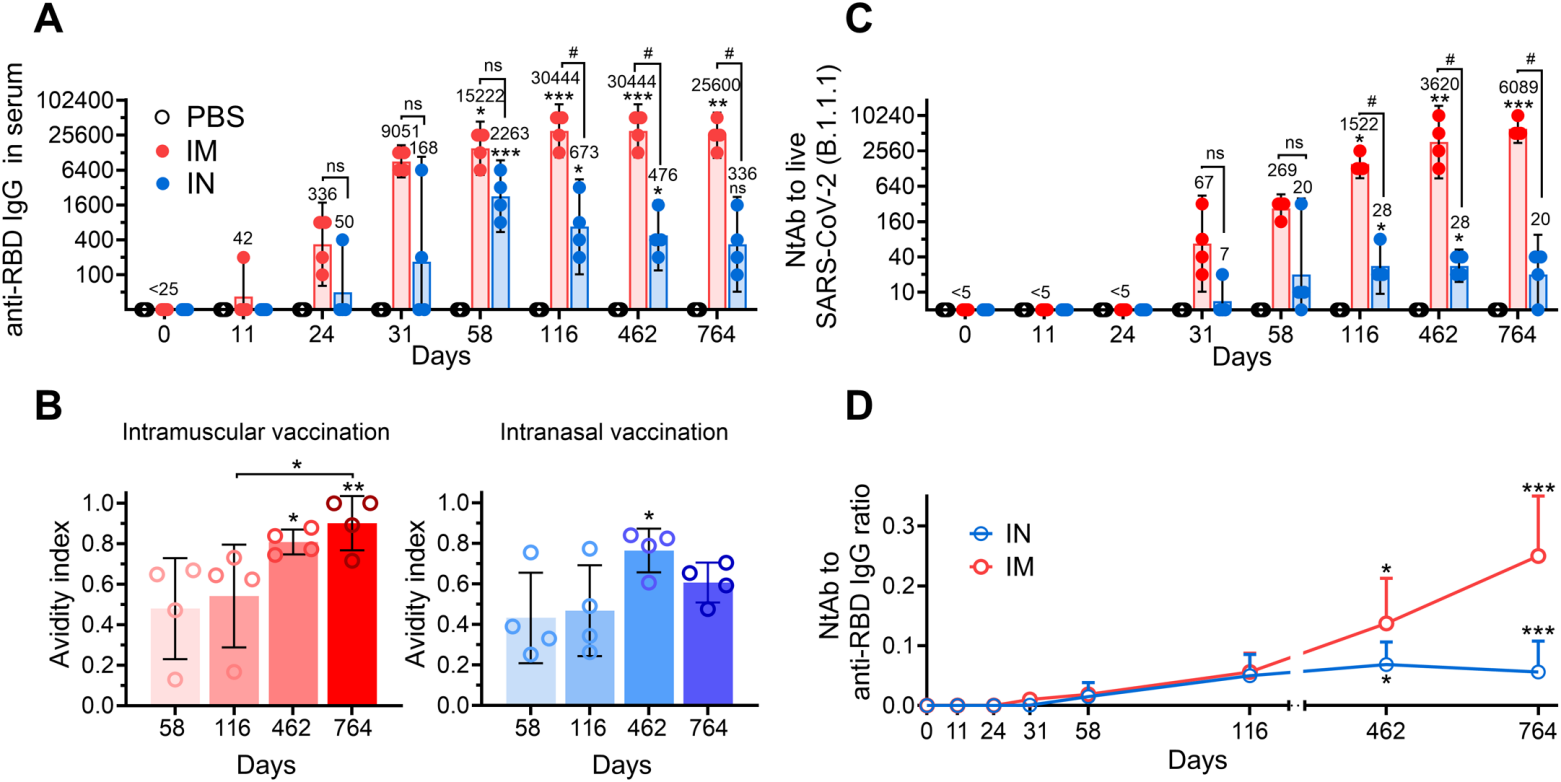
The long-term systemic humoral immune response in common marmosets after intramuscular (IM) or intranasal (IN) vaccination. Time-dependent changes in anti-RBD IgG titers **(A)**, avidity indices of anti-RBD IgGs **(B)** and live (B.1.1.1.) virus-neutralizing antibody (NtAb) titers **(C)** in the serum of common marmosets that IM or IN received the same doses of Sputnik V vaccine. The placebo group received PBS via both the IM and IN route on the same days. Empty circles represent individual data points. Bars represent either geometric mean **(A, C)** or median **(B)** for each group with 95% CI **(A, C)** or SD **(B)**. GMRTs are indicated above each group. Time-dependent changes in anti-RBD to NtAb ratios (the neutralization potency index) in the serum of common marmosets that received the same doses of Sputnik V vaccine via the IM or IN route **(D)**. Empty circles represent the mean values. Whiskers are SD. Significant differences between chosen day and day 0 (before vaccination) are indicated by asterisks (*p<0.05, **p<0.005, ***p<0.001, non-parametric Friedman test with Benjamini, Krieger and Yekutielli correction). The difference between the IM and IN groups on the same day is shown with hashes (#, p<0.05, two-way ANOVA with Tukey’s *post hoc* test). NS, not significant.

A comparative analysis of the long-term systemic antigen-binding antibody response following IN and IM vaccination of NHPs showed similar curves, reaching maximum within 2 years, with a subsequent declining trend (**Figure 2A**). However, a number of distinctive features were observed between studied groups. First, the titers of anti-RBD IgGs were generally higher at all time points in the IM group compared to those in the IN group, but differences became statistically significant only starting from day 116 (GMRT 30,444 vs 673) and lasted up to day 764 (GMRT 25600 vs 336). Second, the peak of anti-RBD IgGs was observed on day 58 (GMRT 2263) upon IN vaccination, while IM vaccination led to delayed and prolonged maximum of antigen-binding response (GMRT 30,444 on both days 116 and 462). Third, at the latest time point (day 764), the IN group experienced significant antibody waning (GMRT 336 became statistically insignificant in the IN group compared to that in the PBS-treated group), while the IM group showed only an insignificant decrease (GMRT 30,444 on day 462 compared to 25,600 on day 764).

Antibody avidity is one of the important parameters indicating the antibody maturation process. It was diversely shown that the antigen-binding strength of antibodies increases with time following both SARS-CoV-2 infection and vaccination (3, 5, 19, 20). Here, we determined changes in serum avidity between yearly and late time points upon IM and IN vaccination. The avidity indices (AI) of anti-RBD IgG in sera from IM vaccinated NHPs showed a continuous increase, reaching the maximum mean value of 0.90 on day 764 (**Figure 2B**). Notably, at the latest time point, sera of two out of four animals demonstrated equal raw and 8M urea-treated OD_450_ values (AI=1). IN vaccination also resulted in an increase in the AI but was lower than that in the IM group on day 462 (0.76 vs 0.81 in the IN and IM groups, respectively) and subsequently decreased to 0.61 by day 764.

Neutralizing antibodies play an important role in immune defense against SARS-CoV-2 infection, thus being a correlate of protection against COVID-19 (21). In this regard, at the first step, we evaluated kinetics of serum neutralizing antibodies (NtAb) against live B.1.1.1 (S: D614G) SARS-CoV-2 virus closely related to vaccine-based Wuhan-Hu-1. IN vaccination resulted in rather low NtAb levels, with only the mean titers on days 116 and 462 (both GMRT were 28) being statistically different from those in the PBS-treated group (**Figure 2C**). IM vaccination resulted in significantly higher mean NtAbs titers starting from day 116 (GMRT 1522), with a continuous increase until the end of the observation period on day 764 (GMRT 6089).

Different trends in IgGs and NtAbs observed in IM and IN vaccinated NHPs prompted us to evaluate the NtAbs/RBD-specific IgG ratio (the serum neutralization potency index), which is considered to be another parameter demonstrating maturation of humoral response and having its impact on the effectiveness of immune resistance to COVID-19 infection (22, 23). Both groups of vaccinated animals showed progression of the NtAbs/IgG ratio in the course of time with some distinctive features (**Figure 2D**). The IM group showed a constant increase in the NtAbs/RBD-specific IgG ratio up to day 764, reaching the highest mean value of 0.25 at the furthest timepoint. The neutralization potency index in the IN group reached its maximum on day 462 (mean value 0.07), with a subsequent slight decrease to 0.06 on day 764, which was still statistically significant (p<0.01) compared to the early time point (day 11).

### 3.3 IM but not IN vaccination results in a positive time-dependent correlation between antibody titers and humoral immunity maturation parameters

Having noticed prominent differences in anti-RBD IgG and NtAb titers, the AI of RBD-specific IgGs and the serum neutralization potency indices between early and late time points in IM and IN vaccinated NHPs, we conducted time-dependent correlation analysis (using serum samples collected on days 58, 116, 462 and 764) to identify the relationship between the measured humoral immunity parameters related to time and the vaccination route.

**Figure 3:**
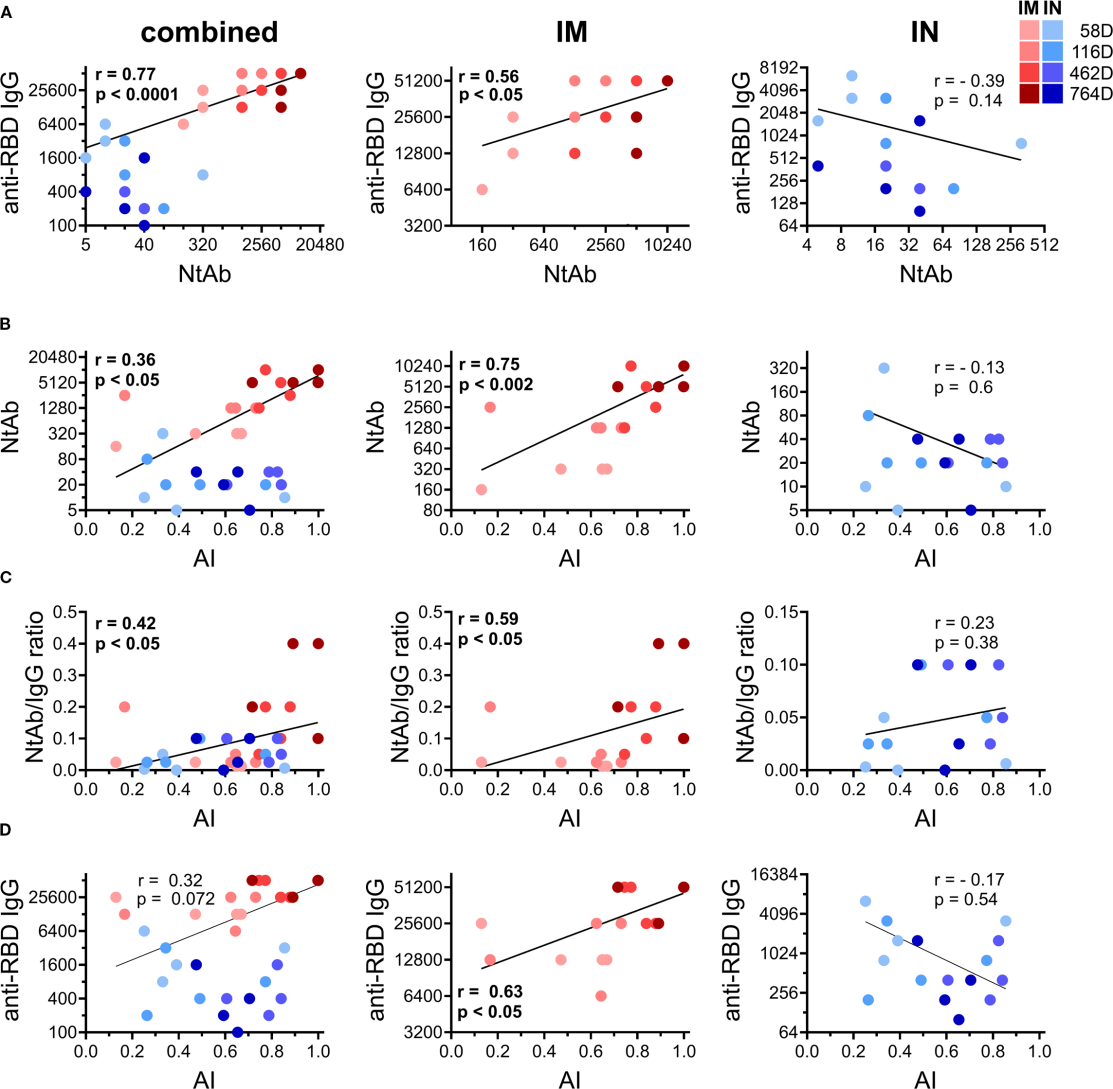
Time-dependent correlations between different humoral immune response parameters measured on days 58, 116, 462, and 764 in all the vaccinated common marmosets (left column) or divided according to the immunization route: IM (middle column) and IN (right column). Log-log summarized correlation plots between titers of anti-RBD IgG and NtAb against live B.1.1.1. SARS-CoV-2 strain in serum of vaccinated NHPs **(A)**. Log-linear correlation plot between NtAb against live B.1.1.1. SARS-CoV-2 strain and avidity indices (AI) of anti-RBD IgG in serum of vaccinated NHPs **(B)**. Linear-linear correlation plot between correlation with NtAb/anti-RBD IgG ratio and the AI of anti-RBD IgG in serum of vaccinated NHPs **(C)**. Log-linear correlation plot between anti-RBD IgG and the avidity indices (AI) of anti-RBD IgG in serum of vaccinated NHPs **(D)**. Color intensity indicates different time points after vaccination (depicted on the legend). The regression line, Spearman non-parametric r and p values are shown (meaningful correlations are indicated in bold values).

We observed that IM and IN vaccination resulted in distinct correlation patterns between all the pairs: RBD IgG and NtAb titers (**Figure 3A**), NtAb titers and the AI of RBD-specific IgGs (**Figure 3B**), the serum neutralization potency index and AI of RBD-specific IgGs (**Figure 3C**), as well as titers and the AI of RBD-specific IgGs (**Figure 3D**). Significant positive correlations between all the pairs were seen upon IM vaccination, which is consistent with the previous findings (4, 24, 25). In contrast, the IN group showed no correlation in any pair. It is also important to note that values of all the correlated parameters were rising in a pairwise manner over time in the IM group, but not in the IN group. These results highlight the differences in the immune response maturation process following IN and IM vaccination.

### 3.4 Intramuscular immunization of NHPs with Sputnik V vaccine results in a significant long-term increase in serum cross-neutralizing activity against SARS-CoV-2 variants

**Figure 4:**
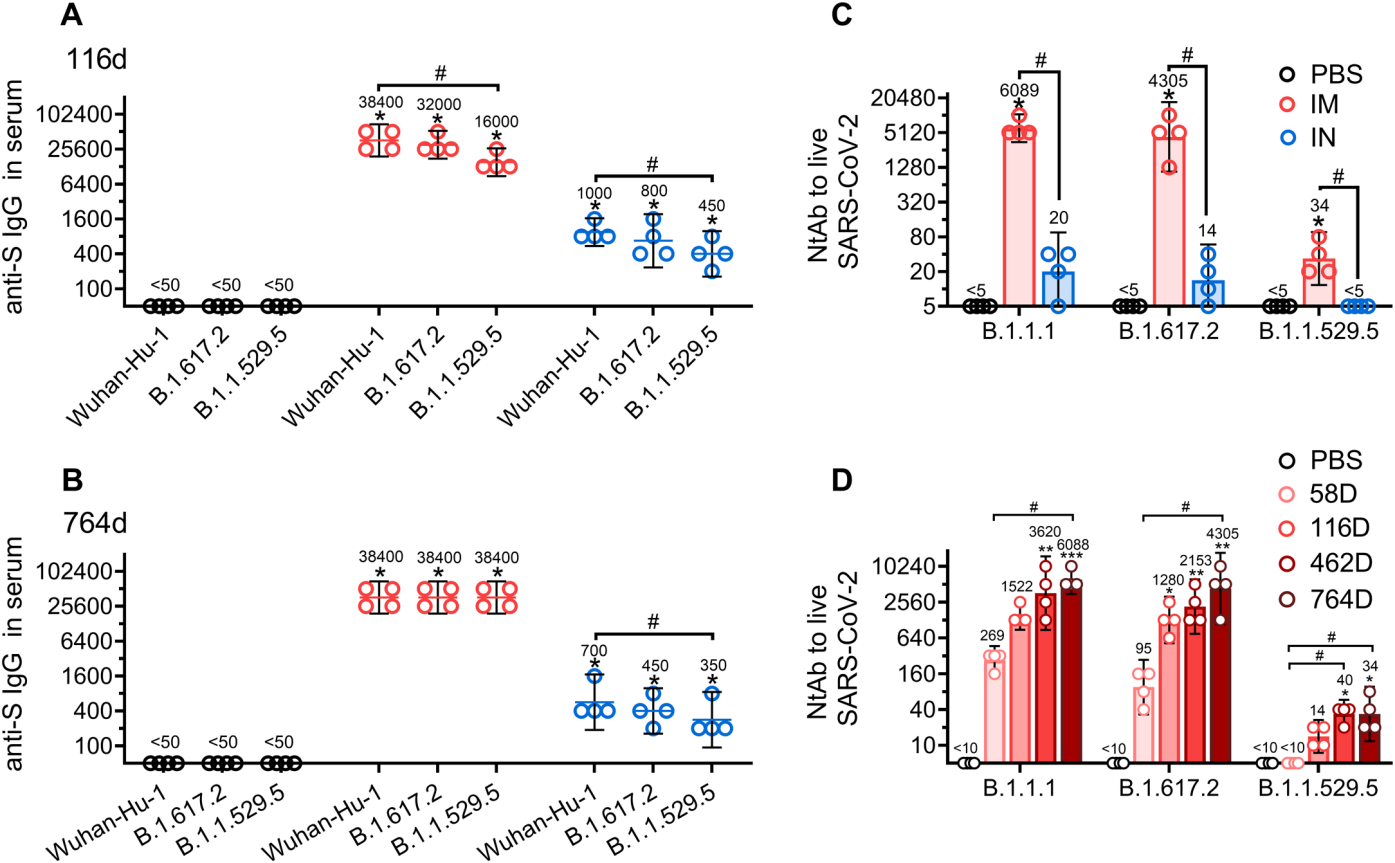
Time-dependent changes in cross-reactivity of serum IgGs and NtAbs in common marmosets that received the same doses of Sputnik V vaccine via the intramuscular (IM) or intranasal (IN) route. IgG titers in sera collected from common marmosets that received, via the IM or IN route, the same doses of Sputnik V vaccine on days 116 **(A)** and 764 **(B)** against S proteins of SARS-CoV-2 Wuhan-Hu-1, B.1.617.2 (Delta) and B.1.1.539.5 (Omicron BA.5) strains. Virus-neutralizing antibody (NtAb) titers against live B1.1.1, B.1.617.2 (Delta) and B.1.1.539.5 (Omicron BA.5) strains of SARS-CoV-2 in sera collected on day 764 from common marmosets that received the same doses of Sputnik V vaccine via the IM or IN route **(C)**. Kinetics of NtAb titers against live B1.1.1, B.1.617.2 (Delta) and B.1.1.539.5 (Omicron BA.5) strains of SARS-CoV-2 in sera obtained from common marmosets IM vaccinated with Sputnik V vaccine **(D)**. The placebo group received PBS via both the IM and IN route on the same days. Empty circles represent individual data points. Lines **(A, B)** or bars **(C, D)** represent the geometric mean for each group with 95% CI. GMRTs are indicated above each group. Significant differences between different time points within one group are shown with asterisks (*p<0.05, **p<0.01, ***p<0.001 paired non-parametric Friedman test). Hashes indicate significant differences between different groups (# p< 0.05, unpaired non-parametric Mann–Whitney U test).

In order to investigate the effect of IM or IN delivered prime-boost Sputnik V vaccine on serum cross-reactivity, we initially evaluated the titers of antigen-binging antibodies against the S proteins derived from vaccine-homologous strain Wuhan-Hu-1, and heterologous Delta (B.1.617.2) and Omicron (B.1.1.529.5) variants of SARS-CoV-2 (**Figures 4A, B**). Using serum samples collected from NHPs on days 116 and 764, we found that the titers of IgGs specific to the S protein from Wuhan-Hu-1 strain were stable over time in the IM group (GMRT 38,400 on both days 116 and 764), while decreasing non-significantly in the IN group (GMRT 1000 vs 700 on days 116 and 764, respectively). Having compared cross-reactivity breadth of IgG antibodies, we found that IgG titers against distanced strains B.1.617.2 and B.1.1.529.5 on day 116 were lower compared to those against the parental strain in both IM (GMRT 32,000 and 16,000, respectively) and IN groups (GMRT 800 and 450, respectively). On day 764, anti-S IgG titers against B.1.617.2 (GMRT 450) and B.1.1.529.5 (GMRT 350) in the IN group still were lower than those against Wuhan-Hu-1 (GMRT 700), while in the IM group, IgGs against both variants (B.1.617.2 and B.1.1.529.5) became equal to the titers against homologous strain (all GMRT 38,400). AI of serum IgG titers against S-proteins from SARS-CoV-2 variants: B.1.1.1, B1.617.2 and B.1.1.529.5 showed a slight (not statistically significant) increase from day 116 to 764 in IM group. IN group showed lower AI values on day 116 compared to IM group, with no AI increase against distanced B.1.617.2 and B.1.1.529.5 in the course of time (**Supplementary Figure S2**).

Evaluation of NHP serum cross-neutralizing activity at the end of the observation period (day 764) showed that IM vaccination resulted in significantly (> 100-fold) higher NtAb titers both against the homologous B.1.1.1 and the heterologous B.1.617.2 and B.1.1.529.5 SARS-CoV-2 strains compared to the IN route (**Figure 4C**). As shown earlier, NtAb titers against the phylogenetically distant heterologous B.1.617.2 and B.1.1.529.5 strains in both the IM and IN groups were lower than titers of NtAbs against the ancestral B.1.1.1 strain (26). On day 764, NtAbs against B.1.1.529.5 (Omicron BA.5) strain (which was not circulating at the time of vaccination) were detected in all animals in the IM group (GMRT 34), whereas being undetectable in the IN group.

Since only IM vaccination resulted in detectable NtAb titers against all the tested SARS-CoV-2 strains at the end of the observation period, we examined the kinetics of serum cross-neutralizing activity from day 58 to day 764 after IM vaccination (**Figure 4D**). Interestingly, NtAb titers increased continuously over time not only against the homologous strain B.1.1.1 (starting from GMRT 269 on day 58 and reaching 6088 on day 764), but also against the heterologous strains B.1.617.2 (from GMRT 95 on day 58 to 4305 on day 764) and B.1.1.529.5 (from undetectable levels on day 58 to GMRT 34 on day 764).

To summarize, both IN and IM vaccination with Sputnik V vaccine induces serum maturation in NHPs, although the immunization route is shown to greatly affect the magnitude and the kinetics of measured parameters.

## 4 Discussion

The sudden spread of the newly emerged SARS-CoV-2 virus, followed by the introduction of multiple COVID-19 vaccines based on different platforms and delivered by different routes and in different doses, has raised a plethora of questions for immunologists to address. Among those, the long-term immunogenicity of COVID-19 vaccines remains one of the least explored areas.

Several parameters of humoral immunity (anti-RBD/S IgG titers, NtAb titers, and antibody affinity/avidity) have been shown to be surrogate markers of protection against COVID-19 [ (21, 27–30)]. Along with that, some of these parameters, such as avidity, neutralization potency, and breadth of serum antigen-binding antibodies against SARS-CoV-2, are shown to be improving over time after vaccination or infection as a result of antibody affinity maturation process (6). In this context, assessment of the long-term immunogenicity of COVID-19 vaccines is highly demanded for vaccine development as well as for planning vaccination campaigns aimed at durable protection of the population. On the other hand, it appears to be a challenging task due to the significant time, financial and labor costs of related research. Constant circulation of SARS-CoV-2 variants in human population, as well as the ongoing massive (re-) vaccination, additionally complicate the dissection of vaccine-induced long-term humoral immunogenicity of COVID-19 vaccines in long-term immunogenicity studies. For instance, our recently published results of phase I-II open, prospective, two-stage, non-randomized clinical study assessing the long-term immunogenicity of Sputnik Lite vaccine showed that SARS-CoV-2 infection greatly affects the kinetics of humoral immune response parameters after vaccination (31).

In order to specifically assess vaccine-induced long-term immune responses while reliably excluding unregistered immune boosts, we performed the experiments in naïve common marmosets (*Callithrix jacchus*, CM), which are naturally nonsusceptible to SARS-CoV-2 infection (13). We have previously demonstrated that both intramuscular and intranasal administration of adenovirus vector-based Sputnik V vaccine induced prominent immune response against SARS-CoV-2 in CMs up to day 116 (14). Here, we monitored the parameters of mucosal and systemic humoral immune response in the same animals over a period of more than two years in order to characterize the long-term maturation of post-vaccination immune response under controlled conditions.

Whenever immune response is studied in animal models, the question regarding clinical relevance of the obtained data must be considered. Here, we demonstrate that intramuscular vaccination of NHPs with Sputnik V leads to a continuous increase in serum antibody avidity and cross-variant neutralization potency over time, which is in line with the findings reported in a number of published longitudinal clinical studies (3, 5, 32). In contrast, in mice, which are widely used for primary assessment of immunogenicity and protectivity of candidate vaccines, the kinetics of antibody avidity upon intramuscular administration of Sputnik V vaccine are markedly different from those obtained in primates (including humans). While the titers of anti-RBD IgGs remained stable between days 77 and 134, the AI of anti-RBD IgGs peaked between days 35 and 49, followed by a prominent decrease by day 91 (**Supplementary Figure S3**). The observed species-specific differences might be caused by, but are not limited to, a shorter lifetime expectancy of mice, a fold difference in biochemical speeds (33), and a significant difference in the number of B cells (34).

One distinctive observation in our study is the long-term persistence of anti-RBD IgG levels in NHP sera after IM (but not IN) vaccination, while in humans, vaccine-induced antigen-binding antibodies generally show a decreasing trend over time (7). It was demonstrated that while the total antibody levels wane because of the rapid loss of low-avidity fraction, high-avidity antibodies remain more stable over time (22, 35, 36). As we observed the relatively high AI of anti-RBD IgG in NHPs’ sera upon vaccination together with a strong correlation with anti-RBD IgG titers (**Figure 3D**), this may explain the persistence of anti-RBD IgG in vaccinated animals. Initial high AI of anti-RBD IgGs observed in IM group might be connected with the relatively high vaccine antigen dose chosen for NHP vaccination. Using two doses of Sputnik V vaccine, we also showed that the vaccine dose affects the kinetics of the AI of anti-RBD IgGs in mice (**Supplementary Figure S3**). The greater dose (10^9^ vp) resulted in the higher AI of anti-RBD IgGs compared to the lower dose (10^8^ vp). The data obtained show that the post-vaccination maturation of humoral immune response depends not only on the number of vaccine doses (4, 24), but also on the dose of the vaccine antigen. Still, detailed studies are needed to assess mechanistic ties between the antigen dose and serum maturation effects.

Here, we demonstrate for the first time that intranasal administration of Ad-vectored COVID-19 vaccine induces serum antibody avidity maturation. However, IN vaccination resulted in less prominent and relatively brief increase in serum neutralization potency, as well as an increase in the AI of anti-RBD IgGs compared to the IM group. Due to the low titers of serum NtAbs, it was impossible to evaluate the time-dependent changes in cross-variant SARS-CoV-2 neutralization breadth in the IN group, while titers of cross-reactive anti-S IgGs against all three tested variants (Wuhan, B.1.617.2, and B.1.1.529.5) decreased between days 116 and 764 in contrast to the IM group. Intranasal vaccination induces generally lower systemic immune responses in humans compared to intramuscular vaccines (37), which might be attributed to natural mucosal immune tolerance as well as differences in vaccine distribution when using different immunization routes. Nasopharyngeal-associated lymphoid tissue (NALT), referred to as the tertiary lymphoid organ (TLO), is the primary site for antigen presentation upon intranasal vaccination, while parenteral immunization predominantly targets secondary lymphoid organs such as spleen and lymph nodes (38, 39). As TLOs are unequal to SLOs in terms of their functions, size and complexity, we assume that the observed partial and time-limited improvement of the AI in NHPs might be caused by functional limitations in GC-driven B cell maturation processes ongoing in TLOs and SLOs (40, 41). To prove this hypothesis, mechanistic studies are needed to unravel the reasons behind the instability of mucosal and systemic immune responses upon intranasal vaccination.

When studying the long-term immunogenicity of intranasal vaccines, the choice of robust preclinical models is getting even more important (42). Given differences in the structure and physiology of the upper respiratory tract of rodents and primates, with a fourfold difference in the ratio between the nasal mucosal surface area and the body volume, species-specific features can be observed even in short-term immune responses in mice upon intranasal immunization (43). For instance, intranasal vaccination of mice showed to be effective in mounting robust mucosal as well as systemic immunity against SARS-CoV-2 (44). Furthermore, in our previous study, we detected similar levels of antigen-binding antibodies in mouse sera after intranasal and intramuscular immunization with Sputnik V vaccine, while intranasally vaccinated CMs showed significantly lower anti-RBD and anti-S IgG titers compared to the intramuscularly vaccinated group (14). These findings are consistent with earlier published reports showing that intranasal and intramuscular ChAdOx1 nCoV-19 vaccination results in similar levels of binding Spike SARS-CoV-2 antigen IgGs in hamster sera, while the results of a phase I clinical trial demonstrated a prominent difference in anti-S IgG serum titers between two immunization routes (37, 45). The results of AdCOVID vaccine based on Ad5 vector platform expressing a human codon-optimized gene for the RBD domain could be another example demonstrating that mice are not a clinically relevant model for evaluating the immunogenicity of intranasal vaccines. Having been highly immunogenic and protective in mice, AdCOVID did not induce an adequate immune response in healthy volunteers (46, 47). Therefore, data suggest that in preclinical studies assessing the immunogenicity of intranasally administered vaccines (the long-term one in particular), general conclusions drawn from large-scale experiments in rodents need to be confirmed by targeted experiments in nonhuman primates.

Among NHPs, the choice of CMs for long-term immunogenicity studies might be prompted by several common factors including their small size, cost-effectiveness, and the relative ease of maintenance under laboratory conditions. The proximity of CMs to human physiology and the immune system constitution (48, 49) make CMs an alternative model used in biomedical research, including preclinical testing of the safety, immunogenicity, and efficacy of antiviral vaccines (50–52). However, like for any animal model, species-specific features must be considered (34).

One of the limitations of our study is that we did not address the mechanism of the observed antibody affinity maturation and the expanding cross-variant neutralizing potency of sera. It has been shown that antibody affinity maturation requires an antigen that can persist in CGs over long periods of time (53). For instance, viral proteins and nucleic acids can persist in the gut of SARS-CoV-2 convalescent individuals for months, providing a source of antigen for GCs (54). In our study, the source of the antigen for GCs in CMs remains unknown.

It is known that the dimeric form of IgA present on mucosal surfaces exhibits higher avidity and neutralizing activity compared to its monomeric form, which is predominantly found in serum (55, 56). Due to the limited availability of anti-marmoset antibodies, another limitation in our study was the inability to separately measure the titers of these different IgA forms. In this study, we used anti-IgA antibodies that recognize both forms of antigen-specific IgA in nasal swabs and serum samples. Overall, this study provides first evidence that not only intramuscular but also intranasal vaccination using adenovirus vector-based Sputnik V vaccine induce serum affinity maturation. It is the first study showing that IN vaccination of NHPs results in time-limited but prominent increase in serum maturation parameters (the avidity and neutralization potency indices), which may contribute to the efficacy of intranasal vaccines. We also showed that IM vaccination appears to be more favorable immunization route for mounting long-lasting systemic humoral immunity with continuously growing serum maturation parameters (antibody avidity, neutralization potency and breadth) over a period of two years. Along with overall reduction in the intrinsic pathogenicity of the modern SARS-CoV-2 strains (53), the maturation of the humoral immune response following intramuscular (IM) and intranasal (IN) vaccination data provide a rationale for the decreased severity of observed cases of the SARS-CoV-2 infections, given that the majority of the human population is vaccinated.

## Data Availability

The raw data supporting the conclusions of this article will be made available by the authors, without undue reservation.

## Supplementary material

Supplementary Figure 1 Time-dependent changes in anti-RBD IgG levels normalized to total IgG concentration in common marmosets following intramuscular (IM) or intranasal (IN) vaccination, as measured in nasal swabs collected on day 7, 116 and 764. The anti-RBD IgGs (expressed in mcg/ml) in nasal swabs **(A)**. For the total IgG concentration measurement **(B)**, a fraction of IgG antibodies was purified from blood serum collected from naïve CM using affinity chromatography with protein A resin (Cytiva, USA). This purified fraction was used to calculate linear part of calibration curve between IgG concentration and optical density (OD). Normalized anti-RBD IgG levels (expressed as percentage) were calculated by dividing anti-RBD IgG concentration by the total IgG concentration **(C)**. The bars represent the mean with standard deviation. Dots show individual data points. Significant differences between chosen day and day 7 is shown above the bars with asterisks (*p < 0.05, non-parametric paired Friedman test).

Supplementary Figure 2 Time-dependent changes in avidity indices (AI) of serum IgG against different S-protein variants B.1.1.1 (ancestral), B1.617.2 (Delta) and B.1.1.529.5 (Omicron BA.5) in common marmosets after intramuscular or intranasal vaccination. Serum is collected on day 116 and 764 after first vaccination. Dots represent individual data points. Bars represent mean for each group with SD.

Supplementary Figure 3 Time-dependent changes in humoral immune response in C57BL/6 mice that received different doses of Sputnik V vaccine via the intramuscular route. Mice received prime boost intramuscular doses of Sputnik V vaccine (10^8^ or 10^9^ vp per dose) with a 21-day interval. Stupefied with isoflurane anesthesia, mice were bled using the submandibular vein method. Blood serum samples were collected from the same mice (n = 6) at indicated time points after first injection and used to evaluate anti-RBD IgGs (A) and its avidity indices **(B)**. Dots represent individual data points. Geometric means **(A)** or medians **(B)** are indicated as lines and numbers above each group. Whiskers are 95% CI **(A)** or SD **(B)**. Asterisks indicate significant differences between indicated days or between the chosen day and day 14 (* p<0.05, ** p<0.01, paired non-parametric Friedman test followed by Benjamini, Krieger and Yekutieli procedure). Hashes indicate significant differences between different doses at the same days (# p<0.05, two-way ANOVA with Bonferroni correction).

## References

[1] COVID19 Vaccine Tracker 2022 (2022). Available online at: https://covid19.trackvaccines.org/vaccines/approved (Accessed December 2, 2022).

[2] WHO. COVID-19 vaccine tracker and landscape (2023). Available online at: https://www.who.int/teams/blueprint/covid-19/covid-19-vaccine-tracker-and-landscape (Accessed March 30, 2023).

[3] Hickey TE Kemp TJ Bullock J Bouk A Metz J Neish A. SARS-CoV-2 IgG Spike antibody levels and avidity in natural infection or following vaccination with mRNA-1273 or BNT162b2 vaccines. Hum Vaccin Immunother. (2023) 19:2215677. doi: 10.1080/21645515.2023.2215677, PMID: 37264688 PMC10305493

[4] Bellusci L Grubbs G Zahra FT Forgacs D Golding H Ross TM. Antibody affinity and cross-variant neutralization of SARS-CoV-2 Omicron BA.1, BA.2 and BA.3 following third mRNA vaccination. Nat Commun. (2022) 13:4617. doi: 10.1038/s41467-022-32298-w, PMID: 35941152 PMC9358642

[5] Nakagama Y Candray K Kaku N Komase Y Rodriguez-Funes MV Dominguez R. Antibody avidity maturation following recovery from infection or the booster vaccination grants breadth of SARS-CoV-2 neutralizing capacity. J Infect Dis. (2023) 227:780–7. doi: 10.1093/infdis/jiac492, PMID: 36546706 PMC10044078

[6] Victora GD Nussenzweig MC. Germinal centers. Annu Rev Immunol. (2022) 40:413–42. doi: 10.1146/annurev-immunol-120419-022408, PMID: 35113731

[7] Collier AY Yu J McMahan K Liu J Chandrashekar A Maron JS. Differential kinetics of immune responses elicited by covid-19 vaccines. N Engl J Med. (2021) 385:2010–2. doi: 10.1056/NEJMc2115596, PMID: 34648703 PMC8531985

[8] Barouch DH Stephenson KE Sadoff J Yu J Chang A Gebre M. Durable humoral and cellular immune responses 8 months after ad26. COV2.S Vaccination. N Engl J Med. (2021) 385:951–3. doi: 10.1056/NEJMc2108829, PMID: 34260834 PMC8314733

[9] Cho A Muecksch F Wang Z Ben Tanfous T DaSilva J Raspe R. Antibody evolution to SARS-CoV-2 after single-dose Ad26.COV2.S vaccine in humans. J Exp Med. (2022) 219:e20220732. doi: 10.1084/jem.20220732, PMID: 35776090 PMC9253517

[10] Wu N Joyal-Desmarais K Ribeiro PAB Vieira AM Stojanovic J Sanuade C. Long-term effectiveness of COVID-19 vaccines against infections, hospitalizations, and mortality in adults: findings from a rapid living systematic evidence synthesis and meta-analysis up to December, 2022. Lancet Respir Med. (2023) 11:439–52. doi: 10.1016/S2213-2600(23)00015-2, PMID: 36780914 PMC9917454

[11] Bates TA Leier HC McBride SK Schoen D Lyski ZL Lee DX. An extended interval between vaccination and infection enhances hybrid immunity against SARS-CoV-2 variants. JCI Insight. (2023) 8(5):e165265. doi: 10.1172/jci.insight.165265, PMID: 36701200 PMC10077480

[12] Edelstein M Wiegler Beiruti K Ben-Amram H Beer N Sussan C Batya P. Vaccine-induced and hybrid immunity to SARS-CoV-2 after three or four doses of BNT162b2 - results from 22 months follow-up of a healthcare workers cohort, Israel, 2020-2022. Int J Infect Dis. (2023) 135:57–62. doi: 10.1016/j.ijid.2023.08.009, PMID: 37572957

[13] Liu Y Hu G Wang Y Ren W Zhao X Ji F. Functional and genetic analysis of viral receptor ACE2 orthologs reveals a broad potential host range of SARS-CoV-2. Proc Natl Acad Sci U.S.A. (2021) 118(12):e2025373118. doi: 10.1073/pnas.2025373118, PMID: 33658332 PMC8000431

[14] Tukhvatulin AI Gordeychuk IV Dolzhikova IV Dzharullaeva AS Krasina ME Bayurova EO. Immunogenicity and protectivity of intranasally delivered vector-based heterologous prime-boost COVID-19 vaccine Sputnik V in mice and non-human primates. Emerg Microbes Infect. (2022) 11:2229–47. doi: 10.1080/22221751.2022.2119169, PMID: 36031930 PMC9518644

[15] Nurmi V Hedman L Perdomo MF Weseslindtner L Hedman K. Comparison of approaches for IgG avidity calculation and a new highly sensitive and specific method with broad dynamic range. Int J Infect Dis. (2021) 110:479–87. doi: 10.1016/j.ijid.2021.05.047, PMID: 34044143

[16] Roy A Saade C Josset L Clement B Morfin F Destras G. Determinants of protection against SARS-CoV-2 Omicron BA.1 and Delta infections in fully vaccinated outpatients. J Med Virol. (2023) 95:e28984. doi: 10.1002/jmv.28984, PMID: 37503561

[17] Sheikh-Mohamed S Isho B Chao GYC Zuo M Cohen C Lustig Y. Systemic and mucosal IgA responses are variably induced in response to SARS-CoV-2 mRNA vaccination and are associated with protection against subsequent infection. Mucosal Immunol. (2022) 15:799–808. doi: 10.1038/s41385-022-00511-0, PMID: 35468942 PMC9037584

[18] Fujimoto C Kido H Sawabuchi T Mizuno D Hayama M Yanagawa H. Evaluation of nasal IgA secretion in normal subjects by nasal spray and aspiration. Auris Nasus Larynx. (2009) 36:300–4. doi: 10.1016/j.anl.2008.09.005, PMID: 19013037

[19] Garcia L Woudenberg T Rosado J Dyer AH Donnadieu F Planas D. Kinetics of the SARS-coV-2 antibody avidity response following infection and vaccination. Viruses. (2022) 14(7):1491. doi: 10.3390/v14071491, PMID: 35891471 PMC9321390

[20] Tauzin A Gendron-Lepage G Nayrac M Anand SP Bourassa C Medjahed H. Evolution of anti-RBD igG avidity following SARS-CoV-2 infection. Viruses. (2022) 14(3):532. doi: 10.3390/v14030532, PMID: 35336940 PMC8949389

[21] Goldblatt D Alter G Crotty S Plotkin SA. Correlates of protection against SARS-CoV-2 infection and COVID-19 disease. Immunol Rev. (2022) 310:6–26. doi: 10.1111/imr.13091, PMID: 35661178 PMC9348242

[22] Moriyama S Adachi Y Sato T Tonouchi K Sun L Fukushi S. Temporal maturation of neutralizing antibodies in COVID-19 convalescent individuals improves potency and breadth to circulating SARS-CoV-2 variants. Immunity. (2021) 54:1841–52 e4. doi: 10.1016/j.immuni.2021.06.015, PMID: 34246326 PMC8249673

[23] Wang M Pan W Xu Y Zhang J Wan J Jiang H. Microglia-mediated neuroinflammation: A potential target for the treatment of cardiovascular diseases. J Inflammation Res. (2022) 15:3083–94. doi: 10.2147/JIR.S350109, PMID: 35642214 PMC9148574

[24] Dapporto F Marchi S Leonardi M Piu P Lovreglio P Decaro N. Antibody Avidity and Neutralizing Response against SARS-CoV-2 Omicron Variant after Infection or Vaccination. J Immunol Res. (2022) 2022:4813199. doi: 10.1155/2022/4813199, PMID: 36093434 PMC9453088

[25] Monroe JM Haralambieva IH Warner ND Grill DE Quach HQ Kennedy RB. Longitudinal antibody titer, avidity, and neutralizing responses after SARS-CoV-2 infection. Heliyon. (2022) 8:e11676. doi: 10.1016/j.heliyon.2022.e11676, PMID: 36439767 PMC9675084

[26] Gushchin VA Dolzhikova IV Shchetinin AM Odintsova AS Siniavin AE Nikiforova MA. Neutralizing activity of sera from sputnik V-vaccinated people against variants of concern (VOC: B.1.1.7, B.1.351, P.1, B.1.617.2, B.1.617.3) and moscow endemic SARS-CoV-2 variants. Vaccines (Basel). (2021) 9(7):779. doi: 10.3390/vaccines9070779, PMID: 34358195 PMC8310330

[27] Hassan AO Case JB Winkler ES Thackray LB Kafai NM Bailey AL. A SARS-CoV-2 infection model in mice demonstrates protection by neutralizing antibodies. Cell. (2020) 182:744–53.e4. doi: 10.1016/j.cell.2020.06.011, PMID: 32553273 PMC7284254

[28] Alsoussi WB Turner JS Case JB Zhao H Schmitz AJ Zhou JQ. A potently neutralizing antibody protects mice against SARS-CoV-2 infection. J Immunol. (2020) 205:915–22. doi: 10.4049/jimmunol.2000583, PMID: 32591393 PMC7566074

[29] Bauer G. The potential significance of high avidity immunoglobulin G (IgG) for protective immunity towards SARS-CoV-2. Int J Infect Dis. (2021) 106:61–4. doi: 10.1016/j.ijid.2021.01.061, PMID: 33713819 PMC7944804

[30] Muecksch F Weisblum Y Barnes CO Schmidt F Schaefer-Babajew D Wang Z. Affinity maturation of SARS-CoV-2 neutralizing antibodies confers potency, breadth, and resilience to viral escape mutations. Immunity. (2021) 54:1853–68 e7. doi: 10.1016/j.immuni.2021.07.008, PMID: 34331873 PMC8323339

[31] Izhaeva FM Tukhvatulin AI Dzharullaeva AS Dolzhikova IV Zubkova OV Shcheblyakov DV. The parameters of long-term humoral immunity induced by a single injection of the sputnik light vaccine among noninfected volunteers and those infected with SARS-CoV-2. Acta Naturae. (2025) 17:52–63. doi: 10.32607/actanaturae.27529, PMID: 40264579 PMC12011181

[32] Lofstrom E Eringfalt A Kotz A Wickbom F Tham J Lingman M. Dynamics of IgG-avidity and antibody levels after Covid-19. J Clin Virol. (2021) 144:104986. doi: 10.1016/j.jcv.2021.104986, PMID: 34563862 PMC8451979

[33] Rayon T Stamataki D Perez-Carrasco R Garcia-Perez L Barrington C Melchionda M. Species-specific pace of development is associated with differences in protein stability. Science. (2020) 369(2020):1449. doi: 10.1126/science.aba7667, PMID: 32943498 PMC7116327

[34] Bjornson-Hooper ZB Fragiadakis GK Spitzer MH Chen H Madhireddy D Hu K. A comprehensive atlas of immunological differences between humans, mice, and non-human primates. Front Immunol. (2022) 13:867015. doi: 10.3389/fimmu.2022.867015, PMID: 35359965 PMC8962947

[35] Ssewanyana I Rek J Rodriguez I Wu L Arinaitwe E Nankabirwa JI. Impact of a rapid decline in malaria transmission on antimalarial igG subclasses and avidity. Front Immunol. (2020) 11:576663. doi: 10.3389/fimmu.2020.576663, PMID: 33584643 PMC7873448

[36] Bullock JL Jr. Hickey TE Kemp TJ Metz J Loftus S Haynesworth K. Longitudinal assessment of BNT162b2- and mRNA-1273-induced anti-SARS-CoV-2 spike igG levels and avidity following three doses of vaccination. Vaccines (Basel). (2024) 12:516. doi: 10.3390/vaccines12050516, PMID: 38793767 PMC11125776

[37] Madhavan M Ritchie AJ Aboagye J Jenkin D Provstgaad-Morys S Tarbet I. Tolerability and immunogenicity of an intranasally-administered adenovirus-vectored COVID-19 vaccine: An open-label partially-randomized ascending dose phase I trial. EBioMedicine. (2022) 85:104298. doi: 10.1016/j.ebiom.2022.104298, PMID: 36229342 PMC9550199

[38] Irvine DJ Aung A Silva M. Controlling timing and location in vaccines. Adv Drug Delivery Rev. (2020) 158:91–115. doi: 10.1016/j.addr.2020.06.019, PMID: 32598970 PMC7318960

[39] Kehagia E Papakyriakopoulou P Valsami G. Advances in intranasal vaccine delivery: A promising non-invasive route of immunization. Vaccine. (2023) 41:3589–603. doi: 10.1016/j.vaccine.2023.05.011, PMID: 37179163 PMC10173027

[40] You X Koop K Weigert A. Heterogeneity of tertiary lymphoid structures in cancer. Front Immunol. (2023) 14:1286850. doi: 10.3389/fimmu.2023.1286850, PMID: 38111571 PMC10725932

[41] Pizzolla A Wang Z Groom JR Kedzierska K Brooks AG Reading PC. Nasal-associated lymphoid tissues (NALTs) support the recall but not priming of influenza virus-specific cytotoxic T cells. Proc Natl Acad Sci U S A. (2017) 114:5225–30. doi: 10.1073/pnas.1620194114, PMID: 28461487 PMC5441821

[42] Carvalho T. Intranasal COVID-19 vaccine fails to induce mucosal immunity. Nat Med. (2022) 28:2439–40. doi: 10.1038/d41591-022-00106-z, PMID: 36329319

[43] Harkema JR. Comparative pathology of the nasal mucosa in laboratory animals exposed to inhaled irritants. Environ Health Perspect. (1990) 85:231–8. doi: 10.1289/ehp.85-1568334, PMID: 2116960 PMC1568334

[44] An X Martinez-Paniagua M Rezvan A Sefat SR Fathi M Singh S. Single-dose intranasal vaccination elicits systemic and mucosal immunity against SARS-CoV-2. iScience. (2021) 24:103037. doi: 10.1016/j.isci.2021.103037, PMID: 34462731 PMC8388188

[45] van Doremalen N Purushotham JN Schulz JE Holbrook MG Bushmaker T Carmody A. Intranasal ChAdOx1 nCoV-19/AZD1222 vaccination reduces viral shedding after SARS-CoV-2 D614G challenge in preclinical models. Sci Transl Med. (2021) 13:eabh0755. doi: 10.1126/scitranslmed.abh0755, PMID: 34315826 PMC9267380

[46] Schultz MD Suschak JJ Botta D Silva-Sanchez A King RG Detchemendy TW. A single intranasal administration of AdCOVID protects against SARS-CoV-2 infection in the upper and lower respiratory tracts. Hum Vaccin Immunother. (2022) 18:2127292. doi: 10.1080/21645515.2022.2127292, PMID: 36194255 PMC9746417

[47] Altimmune Announces Update on AdCOVID™ Phase 1 Clinical Trial (2021). Available online at: https://ir.altimmune.com/news-releases/news-release-details/altimmune-announces-update-adcovidtm-phase-1-clinical-trial (Accessed June 29, 2021).

[48] Gordeychuk IV Gancharova OS Gulyaev SA Gulyaeva TV Zhitkevich AS Avdoshina DV. Experimental use of common marmosets (Callithrix jacchus) in preclinical trials of antiviral vaccines. Acta Naturae. (2024) 16:30–9. doi: 10.32607/actanaturae.27372, PMID: 39188261 PMC11345092

[49] Nelson M Loveday M. Exploring the innate immunological response of an alternative nonhuman primate model of infectious disease; the common marmoset. J Immunol Res. (2014) 2014:913632. doi: 10.1155/2014/913632, PMID: 25170519 PMC4129158

[50] Kozlovskaya LI Piniaeva AN Ignatyev GM Gordeychuk IV Volok VP Rogova YV. Long-term humoral immunogenicity, safety and protective efficacy of inactivated vaccine against COVID-19 (CoviVac) in preclinical studies. Emerg Microbes Infect. (2021) 10:1790–806. doi: 10.1080/22221751.2021.1971569, PMID: 34427172 PMC8439218

[51] Kametani Y Shiina T Suzuki R Sasaki E Habu S. Comparative immunity of antigen recognition, differentiation, and other functional molecules: similarities and differences among common marmosets, humans, and mice. Exp Anim. (2018) 67:301–12. doi: 10.1538/expanim.17-0150, PMID: 29415910 PMC6083031

[52] Moi ML Ami Y Muhammad Azami NA Shirai K Yoksan S Suzaki Y. Marmosets (Callithrix jacchus) as a non-human primate model for evaluation of candidate dengue vaccines: induction and maintenance of specific protective immunity against challenges with clinical isolates. J Gen Virol. (2017) 98:2955–67. doi: 10.1099/jgv.0.000913, PMID: 29160199

[53] Victora GD Nussenzweig MC. Germinal centers. Annu Rev Immunol. (2012) 30:429–57. doi: 10.1146/annurev-immunol-020711-075032, PMID: 22224772

[54] Gaebler C Wang Z Lorenzi JCC Muecksch F Finkin S Tokuyama M. Evolution of antibody immunity to SARS-CoV-2. Nature. (2021) 591:639–44. doi: 10.1038/s41586-021-03207-w, PMID: 33461210 PMC8221082

[55] Waki K Tani H Kawahara E Saga Y Shimada T Yamazaki E. Comprehensive analysis of nasal IgA antibodies induced by intranasal administration of the SARS-CoV-2 spike protein. Elife. (2025) 12:RP88387. doi: 10.7554/eLife.88387, PMID: 40338637 PMC12061477

[56] Chen S Zhang Z Wang Q Yang Q Yin L Ning L. Intranasal adenovirus-vectored Omicron vaccine induced nasal immunoglobulin A has superior neutralizing potency than serum antibodies. Signal Transduct Target Ther. (2024) 9:190. doi: 10.1038/s41392-024-01906-0, PMID: 39039046 PMC11263566

