# Supplementary figures and images for: Immunization route-mediated differences in long-term maturation of humoral immune response induced by adenovirus vector-based COVID-19 vaccine Sputnik V in nonhuman primates
Source: Front Immunol. 2025 Sep 12;16:1634187. doi: 10.3389/fimmu.2025.1634187 (PMC12464043; doi:10.3389/fimmu.2025.1634187)

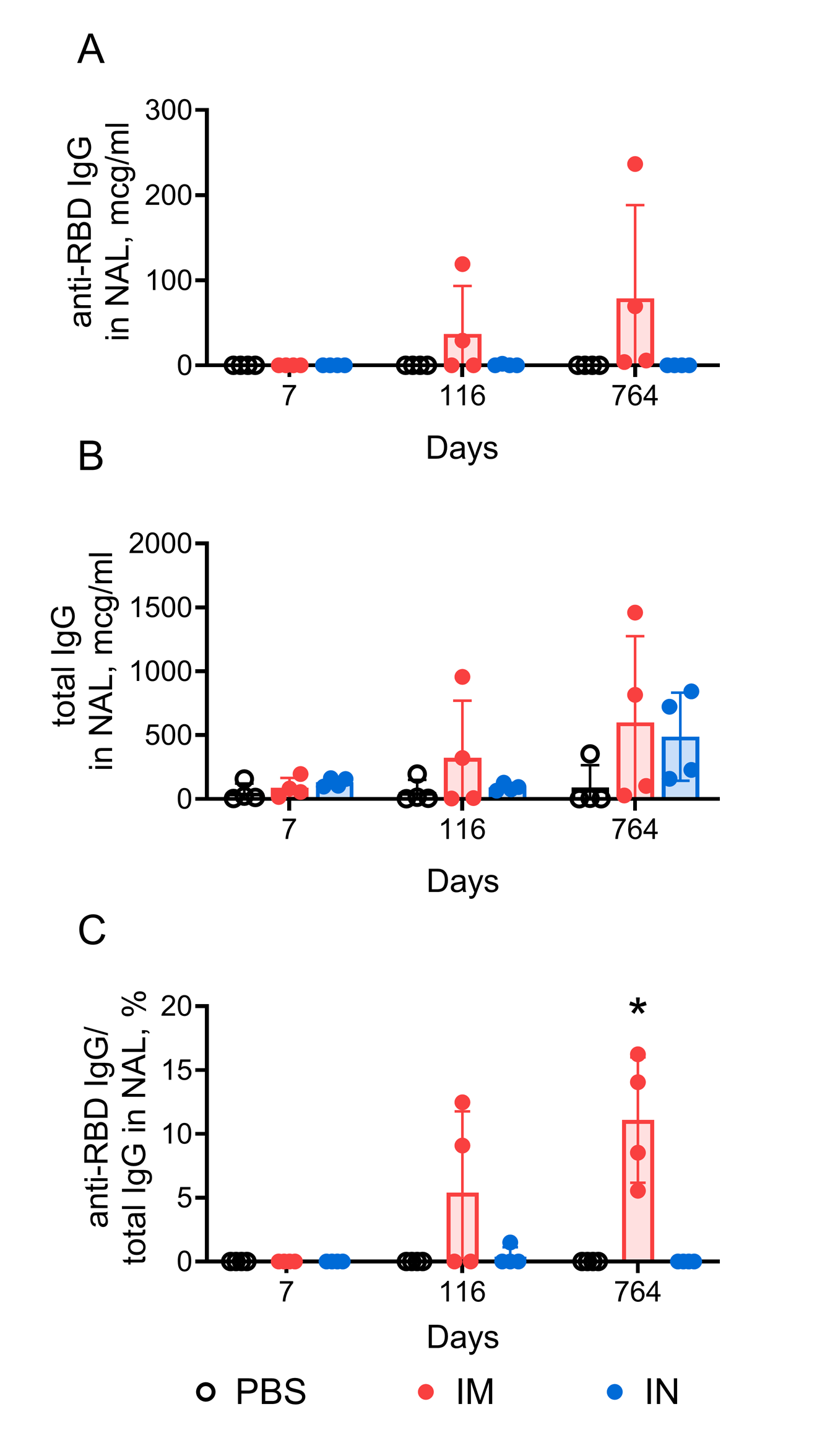

Supplement: Supplementary Figure 1 — Time-dependent changes in anti-RBD IgG levels normalized to total IgG concentration in common marmosets following intramuscular (IM) or intranasal (IN) vaccination, as measured in nasal swabs collected on day 7, 116 and 764. The anti-RBD IgGs (expressed in mcg/ml) in nasal swabs (A). For the total IgG concentration measurement (B), a fraction of IgG antibodies was purified from blood serum collected from naïve CM using affinity chromatography with protein A resin (Cytiva, USA). This purified fraction was used to calculate linear part of calibration curve between IgG concentration and optical density (OD). Normalized anti-RBD IgG levels (expressed as percentage) were calculated by dividing anti-RBD IgG concentration by the total IgG concentration (C). The bars represent the mean with standard deviation. Dots show individual data points. Significant differences between chosen day and day 7 is shown above the bars with asterisks (*p < 0.05, non-parametric paired Friedman test). [file Image1.tif]

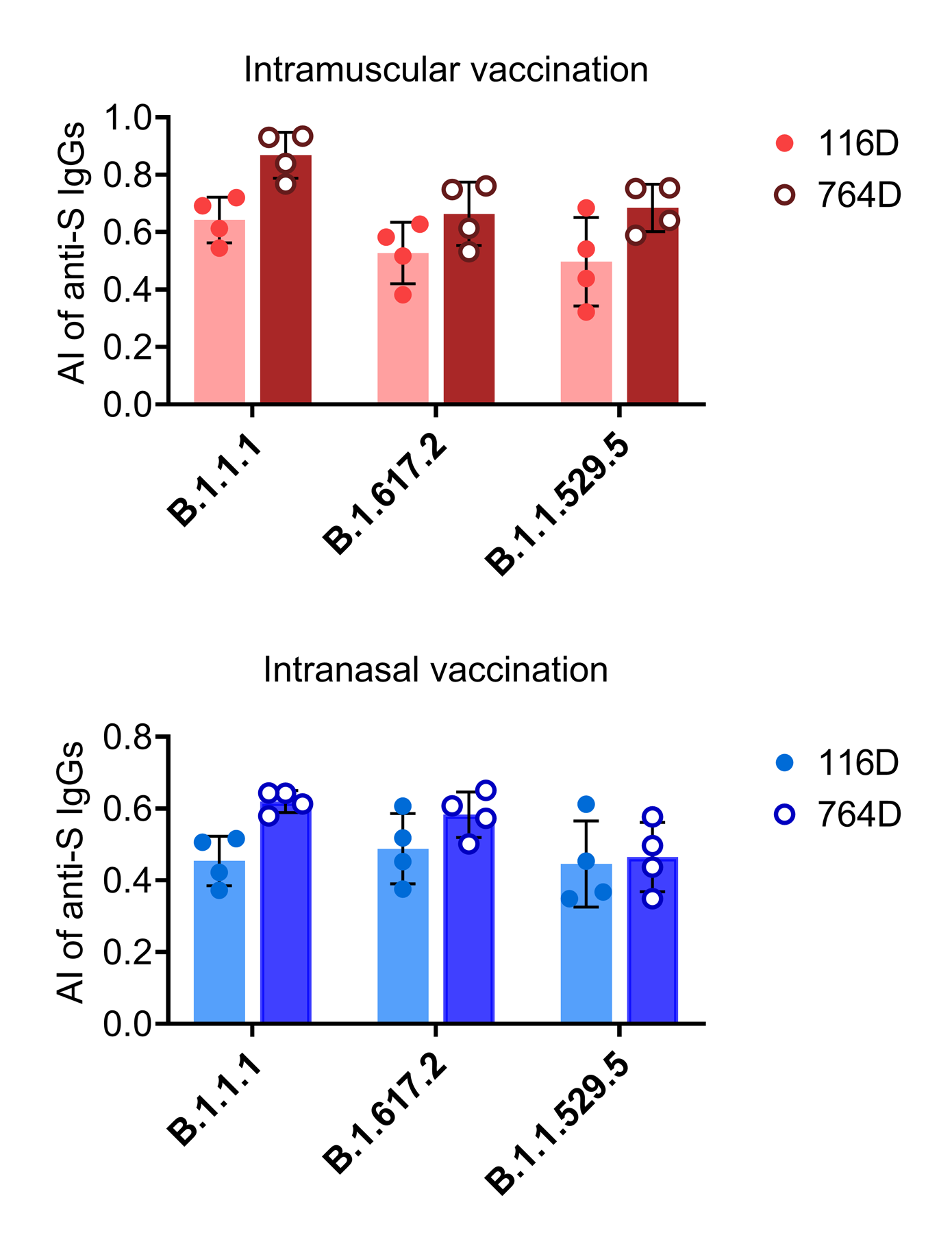

Supplement: Supplementary Figure 2 — Time-dependent changes in avidity indices (AI) of serum IgG against different S-protein variants B.1.1.1 (ancestral), B1.617.2 (Delta) and B.1.1.529.5 (Omicron BA.5) in common marmosets after intramuscular or intranasal vaccination. Serum is collected on day 116 and 764 after first vaccination. Dots represent individual data points. Bars represent mean for each group with SD. [file Image2.tif]

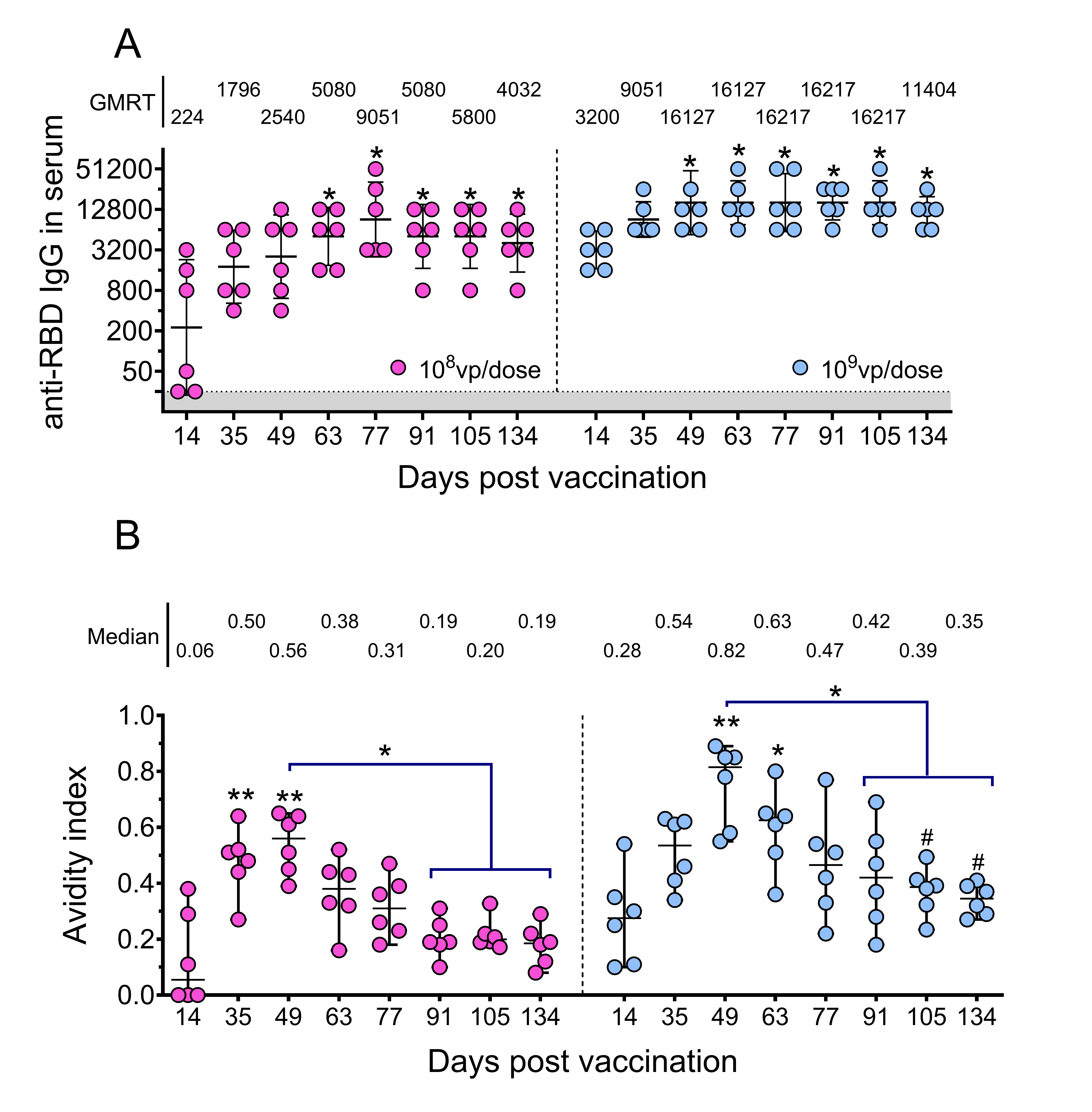

Supplement: Supplementary Figure 3 — Time-dependent changes in humoral immune response in C57BL/6 mice that received different doses of Sputnik V vaccine via the intramuscular route. Mice received prime boost intramuscular doses of Sputnik V vaccine (108 or 109 vp per dose) with a 21-day interval. Stupefied with isoflurane anesthesia, mice were bled using the submandibular vein method. Blood serum samples were collected from the same mice (n = 6) at indicated time points after first injection and used to evaluate anti-RBD IgGs (A) and its avidity indices (B). Dots represent individual data points. Geometric means (A) or medians (B) are indicated as lines and numbers above each group. Whiskers are 95% CI (A) or SD (B). Asterisks indicate significant differences between indicated days or between the chosen day and day 14 (* p<0.05, ** p<0.01, paired non-parametric Friedman test followed by Benjamini, Krieger and Yekutieli procedure). Hashes indicate significant differences between different doses at the same days (# p<0.05, two-way ANOVA with Bonferroni correction). [file Image3.tif]
